# Anesthesia for thoracoscopic surgery

**DOI:** 10.4103/0972-9941.38906

**Published:** 2007

**Authors:** I D Conacher

**Affiliations:** Department of Thoracic Anesthesia, Freeman Hospital, Newcastle Upon Tyne Nhs Hospital Trust, Freeman Road, Newcastle Upon Tyne, NE7 7DN, England

**Keywords:** Analgesia for thoracotomy, lung separators, one lung ventilation

## Abstract

Anesthesia for thoracoscopy is based on one lung ventilation. Lung separators in the airway are essential tools. An anatomical shunt as a result of the continued perfusion of a non-ventilated lung is the principal intraoperative concern. The combination of equipment, technique and process increase risks of hypoxia and dynamic hyperinflation, in turn, potential factors in the development of an unusual form of pulmonary edema. Analgesia management is modelled on that shown effective and therapeutic for thoracotomy. Perioperative management needs to reflect the concern for these complex, and complicating, processes to the morbidity of thoracoscopic surgery.

## INTRODUCTION

Thoracoscopy nearly has a century long history (1912). It is only now that technological advance has enabled move beyond diagnostic and experimental to the therapeutic and even the norm. Most operations, considered as part of the repertoire of the thoracic surgeon and requiring access through a sternotomy or thoracotomy, are within the range of alternative and putatively less destructive thoracoscopic techniques. Initially, operations were conducted with local anesthetic blockade at the point of access, either local anesthetic infiltration or some form of single or extended intercostal nerve blockade (e.g. paravertebral space injection). Hypoxia and accumulation of carbon dioxide occurred as a consequence of a pneumothorax. The mechanism was the phenomenon named ‘pendelluft’ - pendulum air. This described shunting of the physiological dead space between the two lungs during self-ventilation, resulting in a build-up of carbon dioxide and respiratory acidosis; and often was accelerated because of the poor health of patients suffering from infective conditions, notably tuberculosis. Today much of this history still influences the role of anesthesia: pain relief, facilitating instrument access, ensuring gas exchange and dealing with the effects of co-morbidities.

In the background lies a perception, often pressing, that the less apparently invasive the surgery, the more ill the patients who are considered operable. There is no lessening in the complexity of the anesthetic process, the degree of ‘physiological trespass’ or the need for resources to cope with the pain experience. All benefits being, as for surgery, in reduced blood loss, wound pain and hospital stay.

Almost uniquely in surgical practice, anesthetist and surgeon are very much rivals for the same anatomy. Given the proximity of the anesthetist and surgeon in the field, there are pinch points: almost inevitably, conjunction of anesthetic imperative - to oxygenate - will occur with that of surgery not being possible in the presence of lung employed to perform its function.

Dynamic hyperinflation (‘breath-stacking’) during operation and non-cardiogenic pulmonary edema afterwards, rare in other surgical fields, are common enough to be high in the differential of any untoward situations developing perioperatively.

## TRANSITION FROM THE OPEN SURGERY PARADIGM

A certain amount of interdisciplinary re-balancing is necessary as the surgical operative paradigm shifts from open to endoscopic methods. For instance, vascular and gastro-enterological surgeons have to get familiar with new directions of approach to the mediastinum, the oesophagus and the lung apex. They and their anesthetic partners, have to familiarise themselves with the culture, protocol, processes and problems of one lung ventilation (OLV). In turn, the practised need to anticipate that the basket of morbid conditions will expand.

An example is the change in surgical management of myasthenia gravis. Intervention is passing from the traditional sternotomy for thymectomy to a suprasternal mediastinotomy or right video-assisted thoracoscopy.[1] Although the routine of OLV is for muscle relaxants, there is no requirement in myasthenics. Rarely, if ever, is there any requirement for postoperative respiratory support if all neuromuscular blockers, depolarising and non-depolarising, are eschewed. Sensitive use of deep inhalational anesthesia or total intravenous anesthesia and analgesia minimise the hazard of these interval procedures in a life-long slow mutating disease.[2]

## COMMON THEMES OF OLV

It is well within conventional practice to conduct thoracoscopy without formal OLV, but operating risks are increased and less than ideal operating conditions more frequent.[3–5] A requirement for one lung to be shifted away from the operating field largely defines the anesthetic process and is most easily satisfied by deliberately withholding ventilation from one lung - for description the ‘surgical lung’. Operatively, the hemi-thorax becomes surgically more spacious by due process of lung collapse. In some situations this usual sequence may not be sufficient: carbon dioxide insufflation may be required to separate the pleura and maintain collapse.

There are lung ventilation, postoperative management and complication issues specific to OLV.

## ACHIEVING OLV

**Figure 1:**
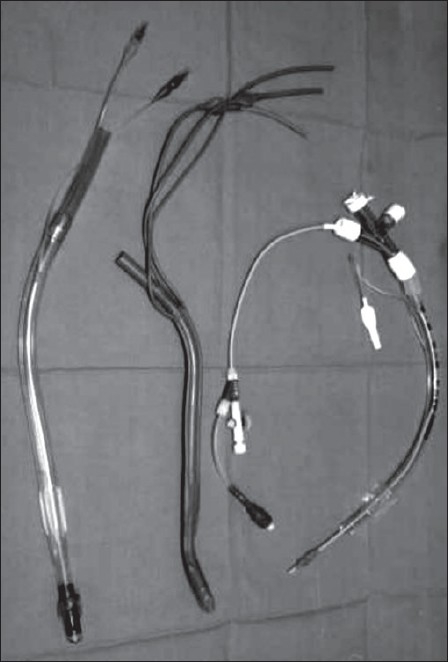
Lung separators. (from L to R) Double lumen tube (right sided - note slot for upper lobe bronchus): Endobronchial tube (Macintosh Leatherdale - left sided): Bronchus blocker (Cohen/Cook model) and ancillary

Lung separators are inserted into the airway and stabilized in one or other bronchus. There are three categories of lung separator: the double-lumen tube, the bronchus blocker and, increasingly difficult to acquire, the endobronchial tube [Figure 1]. For each, there are positives and negatives, which are beyond the brief here. Given the trends in materials and technology and an increasing reliance of the anesthetic corpus on fibreoptic devices to help secure the airway, there is little doubt that the new generation bronchus blockers such as the Univent, Arndt or Cohen cater to perceived modern needs.[6] Logic, that older generation devices (transferable and perfectly adequate) as in many medical consumerist situations, is stifled by the weaknesses for imperatives of commerce and fashion. Modern systems are dependent for safe operation on fibreoptic technology: something of a case of a necessity becoming a virtue. For those resistant to these compelling ideas, it is worth remembering that the double lumen tube is simple, flexible and best sited with endobronchial portion in the left main bronchus.[7] This gives reliably better lung separating conditions and easier change for bilateral and sequential procedures such as sympathectomy or the different phases of an oesophagectomy.[8] It has been noted that the bronchus blockers may not give adequate support to the bronchus of the lung being ventilated with a significant increase in risk of producing air-trapping, circulatory dysfunction and barotraumas.[9] This may be increasingly pertinent with the rise in numbers of the obese in developed societies presenting for surgery. And is just one of several potential causes of a dangerous and confusing clinical complex of dynamic hyperinflation. This may require urgent treatment and a remedial pattern of positive pressure ventilation. The presenting syndromic of hypoxia and hypotension however is much more a common feature when there is intrinisic pulmonary pathology; and is particularly marked in thoracoscopic lung volume reduction surgery.[10,11]

## CONSEQUENCES OF OLV

The effects of stopping the ventilation of one lung can be categorized by the following: some or all of which may result in physiological trespass and can act independently or summate:

- Open pneumothorax and the additive effect of carbon dioxide insufflation
- Increase in anatomical shunt (perfusion of a non-ventilated lung).
- Adoption of the lateral decubitus position

### Open pneumothorax

Once the intrinisic negative pleural pressure dynamic is breached, the lung collapses and retracts toward the hilum due to intrinsic elasticity. So effective usually is the effect of this passive intervention that the additional insufflation of carbon dioxide rarely is required to separate the visceral from the parietal pleura. A lung that does not collapse is as likely to be due to the anchoring by pleural adhesions, as it is a fault of inadequate lung separation. But it is always worth checking the anesthetic side (e.g. inadequate bronchus cuff seal) before seeking and treating pleural adhesions and resorting to carbon dioxide insufflation.

Carbon dioxide creates ‘tension’. The rise in intrathoracic pressure reduces venous return, pulmonary blood flow and increases right heart workload.[5,12] In general and in practice, these would appear to influence process practically no more than occasional and overzealous surgical activity close to the pulmonary artery or atria of the heart.

### Increase in shunt fraction

Stopping ventilation to a lung increases the proportion of the cardiac output that is not oxygenated (shunt fraction). Normally the shunt is in the region of 20-28%, irrespective of whether the anesthetic is volatile agent (isoflurane, sevoflurane, desflurane) or intravenous agent (propofol, opioid) based. Increasing the inspired oxygen realistically does not have any effect above this value. Some patients, notably the younger and fitter tend to the higher level and are harder to deal with. Attendants must resist temptation to raise the level of intervention beyond the safe. Even though it usually reflects a ventilation problem, surgery should not continue until oxygen saturations are consistently above 90%. Attempts to improve the oxygen flux by pharmacological manipulation to produce a ‘pneumonectomy circulation’ should be discouraged.[13] Temporarily ‘soft’ clamping the pulmonary artery to stop the shunt flow is advocated by the experienced, but it too is not without risk that is difficult to justify.

### Adopting the lateral decubitus position

This increases gravitational influences by adding the weight of the mediastinum to other forces that physically reduce the functional residual capacity of the dependent (ventilated) lung. However there may be a beneficial effect as pulmonary blood flow is redistributed to the ventilated zone - in effect improving ventilation/perfusion ratios and reducing the physiological shunt. If bronchus blockers are used, the dependent main bronchus is unsupported and compressed by the weight of the mediastinum: hypoventilation and/or dynamic hyperinflation can result.

### Ventilation of the single lung

It is essential to apply the same minute volume (to maintain normocarbia) to one lung. Given the switch to OLV and to reduce the inflation pressure, it is achieved by reducing the calculated tidal volume to a lower range of 5-10 ml per kg, adjusting the ventilation frequency rate to stabilize the end tidal carbon dioxide.[14]

### Dispute resolution

OLV must never be assumed to be safe.[15] No operating should be conducted with oxygen saturations of less then 90%. The safe position always is ventilation of both lungs and 100% oxygen. A saying ‘CPAP to the top (surgeon' lung) PEEP to the bottom (anesthetist's/dependent lung)’ refers to a traditional view of lateral thoracotomy practice by which some compromise of these conflicting situations can be produced. The oxygen reservoir capacity of the surgeon's lung can be enhanced by partial re-expansion and oxygen insufflation without too much impedance to surgical working conditions.[16] Occasionally, surgery only may be possible by alternating with ventilation; and cycle change instituted as oxygen saturations drop into the low 90's.

## POSTOPERATIVE MANAGEMENT

### Analgesia

Recently the objectives of pain relief have been defined.[17]

1. Humanitarian
2. Reverse the effects of surgery (in context - reduced chest wall and pulmonary compliance)
3. Promote healing
4. Prevent transition to chronic pain syndromes.

Achieving standard for Objectives 1 and 2 is all that is required for much thoracoscopic practice. The painful experience relatively is short-lived; an aided ability to take deep breaths and cough can be anticipated and a rapid return to mobility is likely with early removal of chest drains, if present. Routine and standard patient controlled analgesia systems (PCAS) containing opioids generally are all that are required, in combination with oral supplementation with simple analgesics and non-steroidal anti-inflammatory drugs. Percutaneous paraverteral blockade is known to reduce the pressor reactions to the nociception of thoracic surgery: it is feasible to prolong this form of anti-nociception by inserting epidural-type catheters during surgery and infusion of local anesthetic postoperatively.[18,19] Chest drains also can be used as conduits for local anesthetics. For esphagectomies, in which a more prolonged bedfast and hypercatabolic state is anticipated, resources early should be directed to an epidural-based technique to function throughout the perioperative period. Historical and observational evidence supports the intuitive sense that Objective 3 should be sought to protect and promote healing of anastomoses, increase resistance to infection and the formation of venous thromboses, maintain gastro-intestinal activity and give a sense of well-being during a prolonged debilitating recovery.

There is only speculation that Objective 4 realistically is achievable but a more proactive approach to the design, number and site of ports is likely to pay dividends in the amount of prolonged post-thoracotomy type discomfort and neuralgia.[20]

## POSTOPERATIVE MAINTENANCE INTRAVENOUS FLUID

Special attention to this aspect is necessary because of a condition variously named postoperative pulmonary edema or post-pneumonectomy pulmonary edema. Characterized as a consequence of OLV and classically unilateral, it is part of a spectrum of acute respiratory distress syndrome and as such difficult to separate from more usual confounding diagnoses like pneumonia, bronchiolitis, aspiration and sepsis. Though premorbid dispositions (COPD, cardiac, liver disease), surgical (prolonged handling) and anesthetic (ventilator induced damage) have been identified, prevention policy and management of the condition are best viewed clinically on the basis of a failing or failed pulmonary lymphatic system.[21] Attendants should resort to a traditional colloid fluid restriction (1ml/kg/hour) maintenance policy if any of the specific OLV factors are suspected; and full clinical management upgrade, as for acute respiratory distress syndromes, if signs develop.

## References

[1] Manlulu A, Lee TW, Wan I, Law CY, Chang C, Garzon JC (2005). Video-assisted thoracic surgery thymectomy for non-thymomatous myasthenia gravis. Chest.

[2] Della Rocca G, Coccia C, Diana L, Pompei L, Costa MG, Tomaselli E (2003). Propofol or sevoflurane anesthesia without muscle relaxants allow the early extubation of myasthenic patients. Can J Anesth.

[3] Horswell JL (1993). Anesthetic techniques for thoracoscopy. Ann Thorac Surg.

[4] Rozenberg B, Katz Y, Isserles SA, Baitman B (1996). Near sitting position and two lung ventilation for endoscopic transthoracic sympathectomy. J Cardiothorac Vasc Anesth.

[5] Olsfanger D, Jedeikin R, Fredman B, Shachor D (1995). Tracheal anaesthesia for transthoracic sympathectomy: An alternative to endobronchial anaesthesia. Br J Anaesth.

[6] Cohen H, Benumof JL (1999). Lung separation in the patient with a difficult airway. Curr Opin Anaesthesiol.

[7] Conacher ID (2002). Anaesthesia for thoracoscopic surgery. Best Pract Res Clin Anaesthesiol.

[8] Chui PT, Mainland P, Chung SC, Chung DC (1994). Anaesthesia for three-stage oesphagectomy: An initial experience. Anaesth Intensive Care.

[9] Conacher ID, Velasquez H, Morrice DJ (2006). Endobronchial tubes - a case for re-evaluation. Anaesthesia.

[10] Myles PS, Ryder I (1995). Pulse oximetry. Lancet.

[11] Conacher ID (1998). Dynamic hyperinflation - the anaesthetist applying a tourniquet to the right heart. Br J Anaesth.

[12] Wolfer RS, Krasna MJ, Hasnain JU, McLaughlin JS (1994). Hemodynamic effects of carbon dioxide insufflation during thoracoscopy. Ann Thorac Surg.

[13] Conacher ID (2000). 2000 - Time to apply Occam's Razor to failure of hypoxic pulmonary vasoconstriction. Br J Anaesth.

[14] Gray AJ, Hoile RW, Ingram GS, Sherry KS (1998). Management of patients undergoing oesophagectomy. The Report of the National Confidential Enquiry into Perioperative Deaths 1996/1997.

[15] Pfitzner J, Peacock MJ, Daniels BW (1999). Ambient pressure oxygen reservoir apparatus for use during one-lung anaesthesia. Anaesthesia.

[16] Conacher ID (2006). Pre-emptive analgesia and the paravertebral space: An ignis fatuus. Br J Anaesth.

[17] Kaya FN, Turker G, Basagan-Mogol E, Goren S, Bayram S, Gebitekin C (2006). Preoperative multiple injection thoracic paravertebral blocks reduce postoperative pain and analgesic requirements after video-assisted thoracic surgery. J Cardiothorac Vasc Anesth.

[18] Soni AK, Conacher ID, Waller DA, Hilton CJ (1994). Video-assisted thoracoscopic placement of paravertebral catheters: A technique for postoperative analgesia for bilateral thoracoscopic surgery. Br J Anaesth.

[19] Richardson J, Cheema S (2006). Thoracic paravertebral nerve block. Br J Anaesth.

[20] Slinger PD (2003). Acute lung injury after pulmonary resection:more pieces to the puzzle. Anesth Analg.

[21] Conacher ID (2006). Postoperative pulmonary oedema - tussles with Starlings in the death zone. Anaesthesia.

